# Multimodal perceptual curation for cognitive autonomy in Generation Z decision making

**DOI:** 10.3389/fpsyg.2026.1868429

**Published:** 2026-06-04

**Authors:** Zhean Zhu, Kai Wang

**Affiliations:** 1 School of Data Science, Zhejiang University of Finance and Economics, Hangzhou, China; 2 School of Management, Zhejiang University of Finance and Economics, Hangzhou, China

**Keywords:** algorithmic cognitive colonization, choice architecture, consumption diversity, decision autonomy, dual-process theory, Generation Z, information diet, recommendation algorithm

## Abstract

Recommendation algorithms increasingly shape the perceptual environments through which young users encounter information, evaluate options, and form preferences. Although prior studies have mainly examined algorithmic exposure as a problem of content filtering, less is known about how multimodal digital cues influence perceptual attention, intuitive judgment, and decision autonomy. This study proposes an Algorithmic Cognitive Colonization framework to explain how sustained exposure to personalized recommendation systems may progressively affect attentional selection, preference formation, and self-related decision processes. Two behavioral studies were conducted with educated Chinese undergraduate participants. Study 1 used a 14-day information diet field experiment in which participants switched from personalized recommendation feeds to chronological or social-graph-based feeds while maintaining normal platform use. The intervention was associated with increased reported content-exposure breadth, a U-shaped adjustment pattern in perceived decision autonomy, and a short-term return of content-exposure breadth toward baseline after personalized feeds were resumed. Study 2 used a dual-process decision paradigm to compare rapid consumer choices with deliberative opinion formation. Algorithmic recommendation labels produced stronger conformity in speeded consumer-choice conditions than in more deliberative opinion-formation conditions. Moreover, metacognitive awareness of algorithmic influence was not significantly associated with actual behavioral resistance, supporting a literacy paradox. These findings suggest that personalized recommendation systems may function as perceptual choice architectures that influence digital decision-making primarily by reducing decision friction and guiding intuitive responses. Rather than providing direct neural or physiological evidence, the present studies offer behavioral evidence for how algorithmically curated multimodal environments may shape attention-related choices, state-level preferences, and perceived autonomy. The results, therefore, provide a behavioral foundation for future multimodal neuroscience and perception research using eye-tracking, psychophysiological, neuroimaging, or cross-modal attention measures.

## 1 Introduction

In contemporary digital environments, human perception and decision-making are increasingly mediated by algorithmic recommendation systems. Rather than actively searching for information through search engines, curated directories, or interpersonal recommendations, users now encounter digital content through continuously personalized feeds, ranked interfaces, and platform-generated suggestions. This transformation is particularly consequential for young adults, including Generation Z users, who have grown up in algorithmically curated media ecosystems. Recommendation systems on platforms such as TikTok, YouTube, Instagram, and other short-video or social media applications do not merely determine what information users see; they also shape the perceptual conditions under which attention is allocated, preferences are reinforced, and everyday decisions are made. Prior work on filter bubbles, echo chambers, and epistemic bubbles has demonstrated that algorithmic systems can narrow informational exposure and restructure the boundaries of public knowledge (Zuiderveen Borgesius et al., 2016; Nguyen, 2020; Pariser, 2011). However, the deeper psychological significance of algorithmic curation lies in its potential to alter not only external information access but also internal processes of preference formation, perceived autonomy, and cognitive self-regulation. Understanding this process is, therefore, essential for perception science, human–computer interaction, digital wellbeing, and the broader study of cognitive autonomy in algorithmically mediated societies.

Existing research has provided important insights into algorithmic influence, yet several theoretical and methodological limitations remain unresolved. In recommender systems and consumer behavior research, algorithms are often conceptualized as decision-support tools that predict user preferences, increase click-through rates, or improve perceived usefulness and trust (Chen et al., 2013; Cheung and Masatlioglu, 2021; Shin, 2020). Related studies on automation bias further show that users may over-rely on algorithmic suggestions, even when such suggestions are imperfect or inferior (Banker and Khetani, 2019; Inkpen et al., 2023; Janssen et al., 2020). In response, transparency-based interventions and explainable AI interfaces have been proposed to help users understand, verify, or critically evaluate algorithmic outputs (Erlei et al., 2020; Guo and Zhang, 2025). Meanwhile, studies of digital detox and social media abstinence have examined whether reducing or suspending platform use can improve psychological wellbeing, anxiety, stress, sleep quality, or self-control (Calvert et al., 2025; Coyne and Woodruff, 2023; El-Khoury et al., 2020; Farrukh et al., 2025; Hartanto et al., 2025; Marciano et al., 2023; Radtke et al., 2021; Robertson et al., 2023; Scheppe et al., 2025; Sohail et al., 2024; de Hesselle and Montag, 2024). Despite these advances, existing approaches remain limited in three respects. First, most studies focus on informational exposure rather than the deeper reshaping of preference and autonomy. Second, digital detox paradigms typically confound three mechanisms: reduced content consumption, interrupted social interaction, and removal of algorithmic curation. Third, algorithmic literacy and transparency studies often assume that awareness can translate into behavioral resistance, although young users may understand algorithmic influence while still conforming to algorithmic cues in real time (Dogruel et al., 2022; Felaco, 2025; Perez Vallejos et al., 2021; Zarouali et al., 2021). These limitations suggest the need for a more precise experimental framework that can reduce personalized recommendation exposure while preserving platform access and examine the behavioral pathways through which recommendation cues influence decision behavior.

To address these gaps, this study proposes the Algorithmic Cognitive Colonization framework, which conceptualizes user-facing personalized recommendation environments as dynamic choice architectures that may influence attention, state-level preference expression, and, over longer periods, identity-related autonomy. Drawing on Self-Determination Theory, Dual-Process Theory, and Choice Architecture Theory, the framework argues that algorithmic systems operate not merely as passive filters but as adaptive perceptual environments that continuously organize what users notice, what they prefer, and how they experience self-directed choice (Johnson and Goldstein, 2003; Kahneman, 2011; Peters et al., 2018; Thaler and Sunstein, 2008). Specifically, we theorize three layers of algorithmic influence: Attention-Related Selection, in which platforms structure the informational field of perception; preferential colonization, in which repeated exposure may shape malleable state-level preference expression; and identity colonization, in which long-term reinforcement may eventually affect self-concept clarity and value alignment (Campbell et al., 1996; Cheney-Lippold, 2011; Rouvroy, 2013). Empirically, the present study focuses on the transition from attentional selection to state-level preference expression. Study 1 introduces a 14-day information diet field experiment that reduces exposure to personalized recommendation feeds while preserving normal platform access and social interaction. Study 2 employs a behavioral dual-task paradigm to compare algorithmic-label effects under speeded consumer-choice conditions and more deliberative opinion-formation conditions. Together, these studies examine whether reduced personalized recommendation exposure and acute recommendation cues are associated with changes in reported content-exposure breadth, perceived autonomy, and algorithmic conformity.

In line with the scope of the present Special Issue, we use the term “multimodal” to refer to digitally integrated perceptual inputs, including visual content, textual cues, recommendation labels, social signals, interface affordances, and behavioral traces. However, the present research does not measure neural activation, psychophysiological responses, gaze behavior, or objective audiovisual integration. Accordingly, our aim is not to make direct neuroscientific claims about multimodal processing. Instead, we position the study as a behavioral investigation of how algorithmically curated multimodal environments may reorganize perceived choice conditions and provide a foundation for future neuroscience-oriented research on human–algorithm perception.

This study makes three main contributions.

First, it develops the Algorithmic Cognitive Colonization framework and provides an initial behavioral test of selected Layer 1 and Layer 2 processes. In doing so, it extends prior work on filter bubbles and recommender systems from informational exposure to state-level preference expression and perceived decision autonomy.

Second, it introduces an information diet paradigm that improves upon conventional digital detox designs by reducing exposure to personalized recommendation feeds while preserving platform access and social communication. This design does not fully isolate algorithmic curation from all other platform features, but it offers a more specific behavioral test of personalized recommendation exposure than total abstinence paradigms.

Third, it applies a dual-process perspective to compare algorithmic conformity under speeded consumer-choice conditions and more deliberative opinion-formation conditions. The findings identify task-level behavioral differences in recommendation-label effects, while leaving direct cognitive-load, attentional, and neural mechanisms for future research.

Importantly, the present manuscript does not treat algorithmic curation as a single uniform technical mechanism. Personalized recommendation environments may be implemented through content-based filtering, collaborative filtering, or hybrid architectures. These systems differ in how they infer relevance: content-based systems rely primarily on item features and a user's prior interactions with similar content; collaborative-filtering systems infer relevance from behavioral similarities among users; and hybrid systems combine item-level, user-level, contextual, and engagement-based signals. The present studies do not experimentally separate these architectures at the model level. Instead, they examine personalized recommendation environments as they are experienced by users through visible feed structures, ranking cues, recommendation labels, and default interfaces. Accordingly, the ACC framework should be interpreted as a behavioral framework for user-facing personalized recommendation environments rather than as a claim that all recommender architectures produce identical psychological effects.

The remainder of this paper is organized as follows. Section 2 reviews related work on algorithmic recommendation, decision-making, digital detox interventions, algorithmic literacy, and human–AI interaction. Section 3 develops the theoretical framework by integrating Self-Determination Theory, Dual-Process Theory, and Choice Architecture Theory into the proposed Algorithmic Cognitive Colonization model. Section 4 presents Study 1, including the information diet design, participant recruitment, measures, compliance verification, statistical analyses, and results concerning reported content-exposure breadth, decision autonomy, metacognitive awareness, and short-term post-intervention readjustment. Section 5 reports Study 2, which uses a dual-task experimental paradigm to examine algorithmic conformity across rapid consumer choice and deliberative opinion formation. Section 6 discusses the theoretical, methodological, practical, and policy implications of the findings, as well as limitations and future research directions. Section 7 concludes by highlighting the importance of safeguarding cognitive autonomy in increasingly multimodal and algorithmically curated perceptual environments.

## 2 Related work

The present research draws on two interconnected streams of empirical inquiry that have developed in parallel but remain insufficiently integrated: first, research on how recommendation algorithms shape user decision-making and cognition, and second, experimental studies on the effects of social media abstinence and digital detox interventions. We review the key developments in each stream over the past 5 years, identify their respective contributions and limitations, and demonstrate how the present studies are positioned at their intersection.

### 2.1 Algorithmic recommendation and its effects on user decision-making

A foundational observation in the recommender systems literature is that algorithms do not merely predict pre-existing tastes but may also shape choice behavior. (Cheung and Masatlioglu 2021) developed theoretical models suggesting that recommendations can act as structural anchors that modify aggregate decision-making profiles. (Chen et al. 2013) similarly showed that interaction with recommendation interfaces can influence user preferences through constrained discovery. Collectively, these frameworks support the view that algorithms can function as active choice architectures rather than merely passive information filters, a dynamic relevant to understanding state-level preference formation.

The tendency of users to defer to automated guidance has generated concerns regarding algorithmic autonomy. Extending classical automation bias into contemporary recommendation contexts, (Banker and Khetani 2019) showed that consumers may rely on algorithmic suggestions even when those suggestions are inferior, partly because machine-generated recommendations are perceived as expert cues. This reliance is consistent with rapid heuristic processing, but subsequent empirical studies indicate that human reliance on AI is context-dependent. (Inkpen et al. 2023) observed that in joint human–AI decision-making, users may either under-rely on algorithmic tuning or misinterpret its complementary value. (Janssen et al. 2020) further showed that explainable AI (XAI) interfaces can support decision verification, although experienced decision-makers may still miss subtle machine errors during rapid judgment tasks. (Erlei et al. 2020) identified a notable tension in algorithmic interaction: while stakeholders using algorithmic decision-making (ADM) systems achieve better outcomes when trusting the black-box advice, the introduction of transparency or external evaluation often disrupts fairness perceptions and artificially lowers human cooperation levels. These findings collectively undermine the assumption that simple transparency is an effective antidote to automated cognitive dependency.

A rapidly developing dimension of this debate concerns youth susceptibility and the gap between algorithmic knowledge and behavioral autonomy. Through qualitative explorations of adolescent TikTok users, (Felaco 2025) mapped the algorithmic imaginary, demonstrating that young users develop distinct cognitive and affective awareness of content moderation and recommendation algorithms, often interpreting these systems critically. Consistently, (Guo and Zhang 2025) established that algorithmic transparency significantly boosts platform trust through the mediating role of perceived fairness, an effect positively moderated by the user's algorithmic literacy. However, (Perez Vallejos et al. 2021) exposed the paradox inherent to these systems for youth: while algorithms offer profound benefits in entertainment and streamlined navigation, they simultaneously precipitate profound anxieties regarding privacy, manipulation, and wellbeing. This tension corroborates what we term the literacy paradox: modern youth possess considerable algorithmic literacy and are highly attuned to algorithmic fairness, yet this cognitive awareness routinely fails to translate into active behavioral resistance when interacting with hyper-optimized, high-velocity feeds.

### 2.2 Social media abstinence and digital detox experiments

As public and clinical concern over algorithmic consumption grows, digital detox and platform abstinence have emerged as the predominant behavioral interventions. Systematic longitudinal reviews by (Radtke et al. 2021) and state-of-the-art assessments by (Marciano et al. 2023) establish that these timeouts range from brief daily restrictions to extended total abstinences, frequently yielding measurable psychological dividends. Recent randomized controlled studies have reported similar patterns. (Calvert et al. 2025) found that a 1-week social media detox among young adults was associated with reductions in clinical anxiety and depression, while (Farrukh et al. 2025) reported improvements in stress-related biomarkers among medical students, including reduced cortisol and enhanced heart-rate variability. These physiological and psychological outcomes are consistently echoed across multiple demographics, from undergraduates (El-Khoury et al., 2020) and typical young adults (Coyne and Woodruff, 2023; Sohail et al., 2024) to highly vulnerable developmental populations such as adolescents, who demonstrated reductions in clinical daytime sleepiness following abstinence periods (de Hesselle and Montag, 2024). Qualitative investigations tracing the detox journey underscore a common theme: initiatives are primarily driven by an intrinsic desire for self-reformation and a reclamation of structural control from the platform ecosystem (Scheppe et al., 2025).

However, despite these observed psychological benefits, the implementation mechanisms of digital detox interventions remain limited. Both (Marciano et al. 2023) and (Hartanto et al. 2025) concluded that moderating daily use—such as capping exposure to 30 min—tends to generate more sustainable wellbeing improvements than total digital prohibition, largely due to the severe social costs associated with complete disengagement. This behavioral nuance is further illuminated by (Robertson et al. 2023), who determined that young digital natives do not experience a physiological “craving” for the platform UI itself, but rather an acute necessity for social connectedness and information sharing. Complete abstinence uniquely penalizes this necessity. Addressing this structural tension, researchers have begun investigating more precise interventions. Building on choice architecture theory (Thaler and Sunstein, 2008), digital-nudge research has examined how interface design can shape online behavior without requiring complete platform abstinence (Purohit et al., 2020). Similarly, (Wood and Muñoz 2021) used unplugged assignments to help students reflect on how digital mechanics influence consumer autonomy. Despite these advances, the core detox methodology remains conceptually blunt.

Synthesizing this literature exposes a key methodological limitation that the present research seeks to address. When studies examine the effects of deactivating social media accounts or completely abstaining from platforms, they simultaneously alter content exposure, social interaction, and algorithmic recommendation exposure. As a result, it is difficult to determine whether observed psychological changes are associated with reduced information load, interrupted social connection, or reduced exposure to personalized recommendation architectures. In contrast, the present information diet paradigm narrows the target of intervention by asking participants to reduce their use of personalized recommendation feeds while maintaining platform access and basic social communication. This design does not eliminate all possible confounds, because chronological and social-graph feeds may introduce alternative social cues, and participants may still encounter algorithmically organized content across mobile applications. Nevertheless, it provides a more specific test of reduced personalized recommendation exposure than total abstinence designs.

## 3 Theoretical framework

To integrate the disparate phenomena of personalized recommendation influence into a coherent, testable model, the present research synthesizes insights from Self-Determination Theory, Dual-Process Theory, and Choice Architecture Theory. These theories clarify three interacting dimensions of user-facing personalized recommendation environments: motivational need satisfaction, processing conditions, and interface-level choice architecture. Within this framework, perceived decision autonomy is treated as the focal SDT-related outcome in the present studies, while competence and relatedness are recognized as theoretically relevant boundary conditions that may shape or confound autonomy-related effects. In addition, “algorithmic curation” is not treated as a technically uniform process. Rather, the model focuses on the user-facing recommendation environment through which ranking, sequencing, labels, defaults, and repeated exposure shape perceived choice conditions. This distinction is important because different recommender architectures may produce different psychological effects even when they appear similar at the interface level.

**Figure 1:**
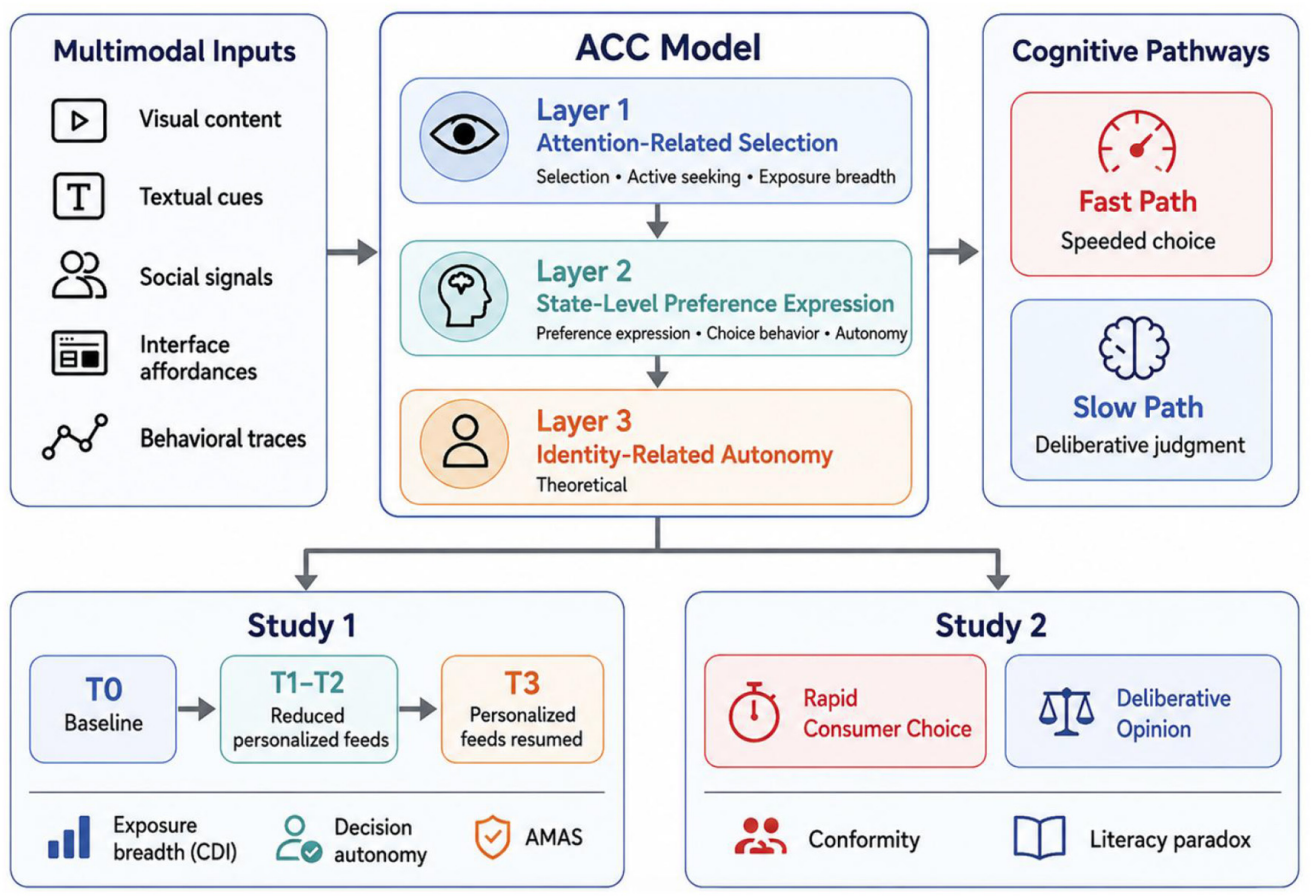
Methodological framework of multimodal perceptual curation for algorithmic cognitive colonization.

Figure 1 presents the overall methodological framework of this study. The framework illustrates how multimodal digital inputs, including visual content, textual cues, social signals, interface affordances, and behavioral traces, are organized within user-facing personalized recommendation environments. These environments are conceptualized as personalized choice architectures that may influence users through selected behavioral layers: attention-related selection, short-term state-level preference expression, and, as a theoretical extension, identity-related autonomy. The figure also summarizes the two empirical studies: Study 1 examines changes in reported content-exposure breadth, perceived decision autonomy, and metacognitive awareness during a 14-day information diet, whereas Study 2 compares recommendation-label conformity under speeded consumer-choice and deliberative opinion-formation conditions.

### 3.1 Self-determination theory and digital need satisfaction

Self-Determination Theory (SDT; Ryan and Deci, 2000) posits that human functioning depends on the satisfaction of three basic psychological needs: autonomy, competence, and relatedness. Autonomy refers to the experience of volition and self-endorsement; competence refers to the sense of effectiveness in navigating one's environment; and relatedness refers to the experience of social connection and belonging. In the context of personalized recommendation environments, autonomy is central to the present research because recommendation interfaces structure the conditions under which users experience digital choices as self-directed. However, feed-structure interventions may also alter competence and relatedness. Switching from personalized feeds to chronological, subscription-based, or social-graph-based modes may increase perceived competence by requiring more active search and selection, while also changing relatedness by altering the visibility of peer-generated content, social cues, and network-based popularity signals.

Accordingly, the present study treats perceived decision autonomy as the focal SDT outcome, but it does not assume that autonomy changes occur independently of competence or relatedness. The information diet intervention may affect perceived autonomy partly through changes in users' sense of effective navigation or social connectedness. This is especially important because chronological and social-graph feeds are not neutral comparison conditions; they may introduce peer salience, recency cues, and network-based visibility. The autonomy trajectory observed in Study 1 should, therefore, be interpreted as a change in perceived self-directed engagement under altered feed architecture, rather than as a pure autonomy effect isolated from the other two SDT needs.

### 3.2 Dual-process theory and algorithmic decision-making

If SDT explains why algorithmic influence matters motivationally, Dual-Process Theory explains how this influence penetrates the cognitive architecture. (Kahneman 2011) popularized the distinction between two modes of thought: System 1, which is often described as rapid, automatic, intuitive, and resource-efficient, and System 2, which is often described as slower, more deliberative, analytical, and resource-intensive. In the present manuscript, this distinction is used as a theoretical lens rather than as a direct measurement claim. Within the context of recommendation systems, the dual-process framework suggests that algorithmic cues may be especially influential when interfaces reduce decision friction and encourage rapid responses. Features such as infinite scrolling, auto-playing videos, and frictionless one-click purchasing may create conditions under which users rely more heavily on intuitive heuristics than on extended deliberation. Recommendation labels can, therefore, operate as salient cues in speeded decision contexts. At the same time, algorithmic information may also shape more reflective judgments when it frames available evidence or preselects arguments for consideration. Study 2, therefore, tests whether recommendation labels produce stronger behavioral effects in speeded consumer-choice tasks than in deliberative opinion-formation tasks, while recognizing that cognitive load, working memory, and response caution are not directly measured.

### 3.3 Choice architecture and algorithmic nudging

The third pillar of our framework concerns the structural environment in which decisions occur. (Thaler and Sunstein 2008) introduced the concept of choice architecture, arguing that the presentation of choices (the order, the defaults, and the framing) inevitably influences the decisions people make. Traditional nudges are static, contextual, and consciously designed interventions (such as placing fruit at eye level in a cafeteria). Recommendation systems extend choice architecture into dynamic and continuously personalized digital environments. First, recommendation interfaces adapt to users' micro-behaviors in real time, making the arrangement of options responsive rather than static. Second, they operate continuously across everyday platform use rather than as isolated decision prompts. Third, they often work through default effects: ranked feeds, top recommendations, preselected content streams, and prominent badges can make some options easier to notice and select than others.

Importantly, recommendation systems rarely operate independently from non-algorithmic interface affordances. Autoplay, infinite scroll, push notifications, haptic feedback, one-click actions, and default feed placement can reduce decision friction and amplify the behavioral influence of recommendation cues. The ACC framework, therefore, treats algorithmic curation and interface affordances as jointly constituting a user-facing personalized choice architecture. The present studies should not be interpreted as estimating the independent causal effect of algorithmic ranking alone; rather, they examine how recommendation cues and personalized feed structures operate within broader friction-reducing interface environments.

### 3.4 Recommendation architecture and the scope of algorithmic curation

Recommendation systems differ in the mechanisms through which they infer relevance and organize exposure. Content-based recommendation systems prioritize items that resemble content with which the user has previously interacted. Their psychological influence may operate primarily through semantic repetition and narrowed topical similarity, gradually reducing exposure to unfamiliar categories. Collaborative-filtering systems infer relevance from the behavior of similar users. Their influence may, therefore, operate not only through content similarity, but also through socially patterned relevance, implicit popularity cues, and perceived alignment with users who are algorithmically classified as similar. Hybrid recommendation systems combine content features, user-similarity patterns, contextual information, and engagement histories. These systems may intensify personalization by merging semantic similarity with behavioral and social inference.

This distinction matters for the ACC framework. Content-based systems may be more likely to support attentional narrowing through repeated exposure to similar content categories, whereas collaborative-filtering systems may contribute more strongly to conformity and social-proof dynamics. Hybrid systems may combine both pathways by simultaneously narrowing topical exposure and reinforcing socially patterned relevance. The present studies do not experimentally compare these architectures. Therefore, ACC should not be read as claiming that all recommender systems operate through identical psychological mechanisms. Rather, it provides a behavioral framework for understanding how user-facing personalized recommendation environments may structure exposure, preference expression, and perceived autonomy. Future studies should directly compare content-based, collaborative-filtering, and hybrid recommendation systems while holding interface features constant.

### 3.5 The Algorithmic Cognitive Colonization (ACC) model

Integrating these insights, we propose the Algorithmic Cognitive Colonization (ACC) model as a layered behavioral framework for describing how user-facing personalized recommendation environments may influence digital perception, information selection, and perceived decision autonomy over time. The model is not intended to collapse content-based, collaborative-filtering, and hybrid recommendation systems into a single technical mechanism. Instead, it focuses on the behavioral level at which different recommendation architectures become visible to users through ranked feeds, default recommendations, recommendation labels, social cues, and repeated exposure patterns. To avoid extending the framework beyond the present empirical design, we distinguish between theoretical layers and operational indicators. The current studies primarily examine short-term, behaviorally observable changes in information selection and state-level preference patterns. They do not provide direct neural, physiological, or eye-tracking evidence of attention, nor do they establish durable changes in trait preferences, identity, or self-concept.

- **Layer 1: Attention-related selection**. At the first layer, user-facing recommendation environments structure the informational field from which users select content. The specific pathway of attentional structuring may differ across architectures: content-based systems may repeatedly foreground semantically similar items, collaborative-filtering systems may foreground items favored by behaviorally similar users, and hybrid systems may combine both forms of exposure narrowing. In the present manuscript, this layer is not operationalized as direct visual attention. No gaze dwell time, scroll depth, fixation pattern, screen-recording, or physiological attention indicators were collected. Instead, Layer 1 is anchored through behavioral proxies of attention-related selection, including exposure to personalized vs. de-personalized feed structures, browser-logged interface use, self-reported active content seeking, and changes in reported content-exposure breadth. These indicators capture how the choice environment redirects selectable content, but they do not establish how long participants visually attended to specific items. Accordingly, claims about Layer 1 should be interpreted as evidence of attention-related behavioral selection rather than direct evidence of visual or neural attention capture.
- **Layer 2: Preferential colonization**. At the second layer, repeated exposure to personalized recommendation environments may shape malleable, state-level preference patterns. The source of this influence may vary by architecture: content-based systems may reinforce topical familiarity, collaborative-filtering systems may reinforce socially patterned relevance, and hybrid systems may reinforce both semantic and social forms of preference expression. In this manuscript, state-level preferences refer to short-term, context-sensitive patterns of content interest that can vary when the surrounding feed architecture changes. They are distinct from trait-level preferences, which are understood as more stable and enduring dispositions. The present study operationalizes selected Layer 2 processes through changes in reported content-exposure breadth, perceived decision autonomy, choice anxiety, and the short-term return of content-exposure breadth toward baseline after personalized feeds were resumed. These indicators support the interpretation that recommendation environments may influence temporary preference expression, but they do not demonstrate that participants' stable tastes or long-term values were altered.
- **Layer 3: Identity colonization**. At the third layer, prolonged algorithmic reinforcement may eventually influence self-concept clarity, value alignment, or identity-related autonomy. This layer is included as a theoretical extension of the ACC model rather than as a directly tested outcome in the present studies. Given the 14-day intervention and 1-week follow-up, the current design cannot establish durable identity change or long-term reconstruction of self-concept. The null findings for self-concept clarity and political attitude extremity are, therefore, interpreted as boundary evidence: short-term changes in feed architecture may affect state-level preference expression without necessarily altering more stable identity-related constructs.

**Figure 2:**
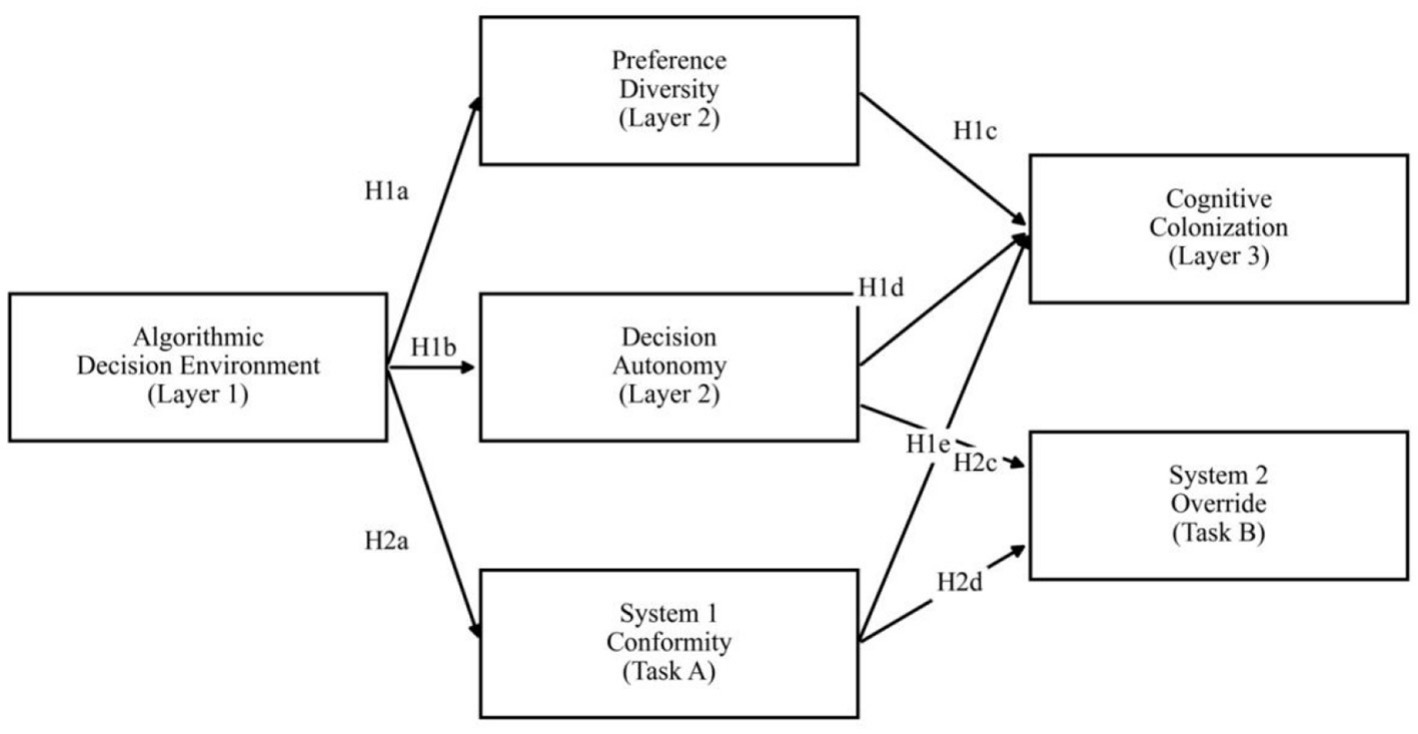
The Algorithmic Cognitive Colonization (ACC) model. The figure illustrates the proposed three-layer structure of algorithmic influence, moving from attention-related selection to short-term state-level preference expression and, as a theoretical extension, identity-related autonomy. In the present studies, only selected Layer 1 and Layer 2 processes are empirically examined through behavioral indicators; Layer 3 remains theoretical and requires longer longitudinal and multimethod evidence.

The conceptual structure of the ACC model is shown in Figure 2. Accordingly, the present research should be understood as a partial empirical test of the ACC framework. Study 1 examines whether reducing exposure to personalized recommendation feeds is associated with short-term changes in reported content-exposure breadth, active content seeking, perceived decision autonomy, and a post-intervention return toward baseline after personalized feeds are resumed. Study 2 examines whether recommendation labels exert stronger influence under speeded consumer-choice conditions than under deliberative opinion-formation conditions. Together, these studies provide behavioral evidence for selected Layer 1 and Layer 2 processes, while Layer 3 remains a theoretical proposition requiring longer longitudinal and multimethod evidence.

### 3.6 Summary of hypotheses

Based on the theoretical framework developed above, we pre-registered the following hypotheses for the two empirical studies.

**Study 1: the information diet experiment**

- **H1a:** Participants in the experimental condition will report a significant increase in content-exposure breadth over the 14-day intervention compared to the control group.
- **H1b:** Decision autonomy in the experimental group will display a U-shaped adjustment pattern, initially declining due to increased decision friction before rising above baseline.
- **H1c:** Algorithmic friction induction will be associated with a statistically significant reduction in political attitude extremity.
- **H1d:** One week following the resumption of personalized recommendation feeds (T3), reported content-exposure breadth in the experimental group will show a short-term return toward baseline levels.
- **H1e:** Metacognitive awareness, as measured by the AMAS, will be significantly higher in the experimental group at post-intervention (T2) compared to baseline (T0).

**Study 2: dual-process decision experiment**

- **H2a:** Algorithmic recommendation labels will significantly increase conformity rates in the speeded consumer-choice task but exert a weaker effect in the deliberative opinion-formation task.
- **H2b:** Providing transparent explanations for recommendations will reduce conformity in the speeded consumer-choice task relative to unexplained recommendation labels.
- **H2c:** Social proof cues will yield higher conformity rates across both decision tasks compared to algorithmic recommendation labels alone.
- **H2d:** Individual differences in cognitive reflection (CRT scores) will moderate the effect of algorithmic labels, such that conformity to recommendation cues will be weaker among high-CRT participants.
- **H2e:** Reflecting the literacy paradox, AMAS scores will be weakly or non-significantly associated with behavioral resistance to recommendation cues in the present task context.

## 4 Study 1: the information diet experiment

Study 1 was a 14-day randomized field experiment designed to examine whether reducing exposure to personalized recommendation feeds was associated with changes in reported content-exposure breadth, perceived decision autonomy, and metacognitive awareness among educated Chinese undergraduate participants. Unlike conventional digital detox paradigms that require complete platform abstinence, the information diet protocol asked participants to switch from personalized recommendation feeds to chronological, subscription-based, or social-graph-based modes while continuing to use their normal platforms. This design reduced personalized recommendation exposure without removing content access or social communication. However, chronological and social-graph feeds may contain their own social-cue structures, and personalized feeds are embedded in broader interface affordances such as autoplay, notifications, infinite scroll, and default feed placement. The intervention is, therefore, best understood as a reduction in exposure to user-facing personalized recommendation environments rather than as a complete isolation of algorithmic influence from other platform design features.

### 4.1 Methods

#### 4.1.1 Participants

A priori power analysis (G^*^Power 3.1) indicated that to detect a moderate interaction effect (*f* = 0.25) in a repeated-measures ANOVA with two groups and four measurements, a total sample of at least 158 participants was required (α = 0.05, power = 0.80). We conservatively recruited 250 undergraduate students from a large university through campus announcements and social media. Inclusion criteria required participants to be aged 18–27 (Generation Z) and to report a minimum daily usage of 1 h on recommendation-driven short-video platforms (e.g., TikTok). Participants already utilizing non-personalized or restricted algorithmic modes were excluded. To ensure analytical integrity, attrition was transparently tracked: 250 participants were initially recruited; 18 failed the baseline inclusion criteria, and 32 were excluded during the 14-day protocol for non-compliance or voluntary withdrawal, resulting in the final analyzed sample of exactly 200 participants (*N* = 100 experimental, *N* = 100 control).

After excluding participants who failed to meet the strict compliance criteria (detailed in Section 4.1.3), the final sample comprised 200 participants (*M*_*age*_ = 20.4, *SD* = 1.8; 62% female). Participants were randomly assigned to either the experimental condition (*N* = 100) or the control condition (*N* = 100). There were no significant baseline differences between the groups regarding age, gender, daily screen time, or primary platform preferences (all *ps* > 0.10). Participant demographics are summarized in Table 1.

**Table 1:** Participant demographics (Study 1).

Variable	Experimental (*N* = 100)	Control (*N* = 100)	*p*-value
Age (*M* ±*SD*)	20.5 ± 1.8	20.3 ± 1.7	0.42
Female (%)	60%	64%	0.56
Daily screen time (hours)	4.2 ± 1.1	4.3 ± 1.0	0.51
Primary platform (TikTok; %)	68%	71%	0.64

#### 4.1.2 Design

The study employed a 2 (Condition: Experimental vs. Control) × 4 (Time: T0, T1, T2, T3) mixed experimental design (with randomized group assignment). Time was the within-subjects factor: T0 served as the pre-intervention baseline, T1 represented the mid-point assessment (Day 7), T2 was the immediate post-intervention assessment (Day 14), and T3 was a delayed follow-up assessment administered 1 week after participants returned to their normal personalized feed use (Day 21) to examine short-term post-intervention readjustment. Condition was the between-subjects factor. The dependent variables included reported content-exposure breadth, decision autonomy, metacognitive awareness, choice anxiety, and political attitude extremity.

#### 4.1.3 The information diet protocol

The core methodological innovation of Study 1 was the implementation of the information diet. This manipulation reduced participants' exposure to user-facing personalized recommendation surfaces, including ranked feeds, recommendation pages, homepage suggestions, and algorithmically organized timelines, without manipulating the underlying recommender architecture implemented by each platform. Accordingly, the intervention should be interpreted as a reduction in personalized recommendation-interface exposure rather than as evidence specific to content-based, collaborative-filtering, or hybrid algorithmic models.

1. Short-Video Platforms (e.g., TikTok): Participants were instructed to exclusively use the Following or Friends tab and strictly avoid the algorithmic For You page. Additionally, they were asked to reset or clear their algorithmic preference cache where possible.
2. Video/Content Platforms (e.g., YouTube): Participants were required to turn off autoplay, pause watch history, clear search history, and navigate using subscription-based or direct-search navigation rather than homepage recommendations.
3. Text/Image Feeds (e.g., Instagram, X): Participants switched from algorithmic timelines to chronological sorting modes.
4. Information/News: Participants were encouraged to use RSS readers (e.g., Feedly) or direct website navigation rather than algorithmically aggregated news feeds.

To evaluate protocol adherence and manipulation validity, the study employed a multi-method verification strategy. First, participants in the experimental group completed brief daily compliance logs via Qualtrics, reporting estimated time spent on personalized recommendation interfaces vs. chronological, subscription-based, or social-graph-based interfaces. Second, participants installed a lightweight, privacy-preserving browser monitoring tool that used local DOM-element detection to differentiate selected platform interface types, such as recommendation-oriented pages vs. following or subscription-based pages. The tool sampled interface exposure at 60-s intervals and recorded only hashed metadata rather than raw content. Third, participants submitted device screen-time screenshots every 3 days to provide an additional check on total platform use.

To address potential compensatory platform switching, the revised protocol also distinguished target-platform compliance from substitution to alternative algorithmically curated platforms. In the daily logs, participants reported whether they increased time on other recommendation-driven services not included in their primary platform list, such as short-video recommendation feeds, algorithmically ranked video pages, or personalized news aggregators. The device screen-time screenshots were reviewed not only for total platform use, but also for changes in major alternative platform categories where such information was available. These indicators were treated as exploratory substitution checks rather than exhaustive behavioral monitoring, because mobile operating systems do not reliably identify whether time within an app was spent on personalized recommendation pages, chronological feeds, direct search, or interpersonal communication.

To assess whether the intervention was experienced as a reduction in personalized algorithmic curation, participants also completed perceived algorithmic disengagement items at T1 and T2. These items asked whether their feeds felt less personalized, less automatically selected, less recommendation-driven, and more dependent on active self-selection during the previous week. Participants who maintained more than 80% compliance across the available compliance indicators received a financial bonus. Because mobile applications restrict the granularity of external behavioral monitoring, this procedure was not intended to capture every instance of in-app exposure or cross-platform substitution. The control group continued their usual digital habits and completed the same daily logs to control for measurement and reporting effects.

#### 4.1.4 Measures

The following measures were administered at all four time points (T0, T1, T2, and T3), unless otherwise specified.

**Consumption diversity index (CDI)**. We developed the CDI to capture the breadth of reported content exposure across predefined content categories. Participants were presented with a catalog of 24 distinct content categories (e.g., domestic politics, international news, comedy, science/tech, fashion, and specialized hobbies). They selected the categories they had consumed over the past seven days. Based on these selections, a Shannon entropy score was calculated [*H* = –∑ *p*_*i*_ ln(*p*_*i*_)], where *p*_*i*_ represents the proportion of selected items. The theoretically maximum possible diversity score for this 24-category index is ln_(24)_ ≈ 3.18. In the revised interpretation, CDI is treated as an exposure-breadth indicator rather than a direct measure of visual attention, intentional exploration, or stable preference change. A higher CDI score indicates that participants reported exposure to a wider range of content categories, but it does not establish how long they attended to specific content or whether the exposure resulted from active selection rather than incidental browsing. For this reason, CDI was interpreted together with daily process-log indicators of active content seeking and choice anxiety.

**Decision autonomy**. To operationalize volition and self-endorsement of choices within digital environments, we adapted the Autonomy subscale from the Basic Psychological Need Satisfaction and Frustration Scale (BPNSFS; Chen et al., 2015). The five-item analog was tailored to the digital context (e.g., “When discovering new digital content, I feel my choices express who I really am”) and rated on a five-point Likert scale. Reliability was high across assessments (α range: 0.82–0.87). Competence and relatedness were not measured as separate dependent variables in the present design; this measurement boundary is considered in the interpretation of autonomy findings and in the limitations.

**Algorithmic metacognitive awareness scale (AMAS)**. To assess users' awareness of algorithmic influence and perceived capacity for resistance, we developed the 12-item AMAS. The scale included four theoretically derived dimensions: algorithm identification, influence awareness, perceived degree of influence, and perceived resistance capacity. Items were rated on a five-point Likert scale from 1 = strongly disagree to 5 = strongly agree. In the revised analyses, we evaluated the scale using internal consistency, confirmatory factor analysis, convergent validity, and discriminant validity. Internal consistency was assessed using Cronbach's α and composite reliability. Convergent validity was examined through standardized factor loadings and average variance extracted, whereas discriminant validity was evaluated by comparing inter-factor correlations and the square root of average variance extracted. Because the present sample was not designed primarily for scale development across demographic groups, measurement invariance analyses should be interpreted cautiously and are reported as exploratory where applicable.

**Choice anxiety**. Adapted from the Maximization Scale (Schwartz et al., 2002), this four-item measure assessed the cognitive friction and anxiety associated with having to actively search for content rather than relying on passive recommendations (e.g., “I feel overwhelmed when I have to actively decide what to read or watch next”). Reliability was acceptable (α = 0.78).

**Political attitude extremity**. Participants rated their position on six polarized policy issues on a 0–100 slider (50 = strictly neutral). Extremity was operationalized as the mean absolute deviation of these six items from the neutral midpoint, yielding a score between 0 and 50.

**Self-concept clarity (SCC)**. The 12-item scale developed by (Campbell et al. 1996) measured the extent to which self-beliefs were clearly and confidently defined (e.g., “In general, I have a clear sense of who I am and what I am”). Study 1 measures are summarized in Table 2.

**Table 2:** Summary of measures (Study 1).

Construct	Items	Reliability (α)
Reported content-exposure breadth (CDI)	24	N/A
Decision autonomy	5	0.82–0.87
Metacognitive awareness (AMAS)	12	0.89
Choice anxiety	4	0.78
Self-concept clarity	12	0.85

#### 4.1.5 Procedure

The study was approved by the Institutional Ethics Review Board. After providing informed consent, participants completed the baseline survey (T0) via Qualtrics and were randomly assigned to conditions. The experimental group attended a brief virtual orientation (via Zoom) standardizing the technical implementation of the information diet protocol. Participants independently executed the protocol for 14 days, providing daily logs. At Day 7 and 14, all participants received automated email links to complete the T1 and T2 surveys, respectively. Following T2, the experimental group was instructed to revert to normal algorithmic usage. On Day 21, the final follow-up survey (T3) was administered. Upon completion, participants were debriefed, asked to report any adverse or disruptive experience during the protocol, and compensated according to their compliance records.

#### 4.1.6 Ethical safeguards for the information diet protocol

The information diet protocol was designed to reduce reliance on personalized recommendation feeds without restricting access to essential information, social communication, or user-initiated search. Participants were not asked to stop using digital platforms, avoid direct searches, disconnect from friends, or refrain from accessing academic, health, safety, or news-related information. Instead, they were instructed to replace personalized recommendation surfaces with chronological feeds, subscription pages, direct-search navigation, RSS readers, official websites, and interpersonal communication channels where possible.

To reduce the risk of informational deprivation, the daily logs allowed participants to report difficulties, discomfort, missed information, or situations in which the protocol interfered with normal academic, social, or informational needs. Participants could discontinue the protocol at any time and still receive prorated compensation. The debriefing procedure also asked participants to report whether the information diet caused meaningful academic, social, or informational disruption. These safeguards were intended to balance the experimental reduction of personalized recommendation exposure with participants' continued access to necessary information and communication.

### 4.2 Results

#### 4.2.1 Preliminary analyses

Compliance monitoring revealed high protocol adherence within the experimental group. Objectively logged de-personalized interface usage via the browser monitoring tool averaged 84.6% of total daily platform time (*SD* = 7.8%), closely corroborating self-reported estimates (*M* = 88.4%, *SD* = 6.2%; agreement *r* = 0.82). Manipulation-check items administered at T1 and T2 further indicated that experimental participants experienced their feeds as less personalized, less automatically selected, less recommendation-driven, and more dependent on active self-selection, supporting the validity of the protocol as a reduction in personalized recommendation exposure. Exploratory substitution checks did not indicate a systematic increase in total screen time or alternative recommendation-driven platform use in the experimental group during the intervention period. Including total screen time as a covariate did not substantively change the Condition × Time patterns for consumption diversity or perceived decision autonomy. Nevertheless, app-level screenshots and browser logs could not fully decompose mobile in-app behavior by feed type; these analyses, therefore, reduce but do not eliminate the possibility of residual in-app exposure or compensatory switching to other algorithmically curated platforms. Independent-samples *t*-tests confirmed no baseline (T0) differences between the experimental and control groups on any primary dependent variables (CDI, decision autonomy, AMAS, choice anxiety, or political extremity; all *p* > 0.15). Missing data (< 2%) were handled using maximum likelihood estimation. Preliminary psychometric analyses supported the proposed four-factor structure of the AMAS (χ^2^/*df* = 1.84, CFI = 0.96, and RMSEA = 0.05). Internal consistency was satisfactory for the full scale and its subdimensions, and additional convergent and discriminant validity checks supported the interpretation of AMAS as a measure of self-reported metacognitive awareness of algorithmic influence. Importantly, AMAS was not treated as a direct behavioral measure of resistance, but as a self-report measure later compared with actual conformity behavior in Study 2.

#### 4.2.2 Main analyses

To test hypotheses H1a through H1e, a series of 2 (Condition) × 4 (Time) mixed repeated-measures ANOVAs were conducted. Where the assumption of sphericity was violated, Greenhouse-Geisser corrections were applied.

**Reported content-exposure breadth (CDI; H1a and H1d)**. The ANOVA for the Consumption Diversity Index (CDI) revealed a significant main effect of Time, *F*_(2.6, 514.8)_ = 10.54, *p* < 0.001, ${\;\eta }_{p}^{2}$ = 0.051, but no significant main effect of Condition. Crucially, as predicted, a significant Condition × Time interaction emerged, *F*_(2.6, 514.8)_ = 12.38, *p* < 0.001, ${\;\eta }_{p}^{2}\;=$ 0.059. Simple main effects analysis clarified this interaction. In the experimental group, CDI scores significantly increased from baseline T0 (*M* = 2.14, *SD* = 0.42) to post-intervention T2 (*M* = 2.48, *SD* = 0.47, *p* < 0.001, and *d* = 0.76), supporting H1a as an increase in reported content-exposure breadth. Conversely, the control group's CDI remained stable across all time points. Addressing H1d, the experimental group's CDI decreased significantly from T2 to the 1-week follow-up T3 (*M* = 2.22, *SD* = 0.43, *p* < 0.001, and *d* = 0.58), indicating a short-term return toward baseline after personalized feeds were resumed. These dynamics provide behavioral evidence consistent with short-term changes in reported exposure breadth: CDI increased when personalized recommendation exposure was reduced and then moved back toward baseline after personalized feeds were resumed. This 1-week pattern should not be interpreted as evidence of durable re-colonization, permanent preference restoration, or stable taste change. Because CDI indexes reported exposure breadth rather than intentional exploration or direct visual attention, it was interpreted alongside the daily increase in active content seeking. This combined pattern suggests broader reported exposure accompanied by greater self-reported active selection, rather than a stand-alone indicator of deeper preference transformation.

**Decision autonomy (H1b)**. The ANOVA for decision autonomy revealed a robust Condition × Time interaction, *F*_(3, 594)_ = 18.62, *p* < 0.001, ${\;\eta }_{p}^{2}$ = 0.086. Trend analysis in the experimental group confirmed a significant quadratic trend, *F*_(1, 99)_ = 25.41, *p* < 0.001, ${\;\eta }_{p}^{2}$ = 0.204, capturing the hypothesized U-shaped adjustment pattern. Experimental participants experienced a significant drop in autonomy from T0 (*M* = 3.65) to T1 (*M* = 3.28, *p* < 0.001), reflecting the friction introduced by reduced personalized recommendation support, followed by a significant rebound above baseline at T2 (*M* = 3.92, *p* = 0.008 vs. T0). This pattern supports H1b, suggesting an initial period of increased decision friction followed by a rebound in perceived self-directed engagement under reduced personalized recommendation exposure. The absence of separate competence and relatedness measures limits this result to perceived autonomy and precludes claims that the other SDT needs remained unchanged.

**Political attitude extremity (H1c)**. Contrary to findings in partial deactivation studies, the Condition × Time interaction for political extremity was marginal and non-significant, *F*_(2.8, 554.4)_ = 2.15, *p* = 0.094, ${\;\eta }_{p}^{2}$ = 0.011. The experimental group demonstrated only a slight, non-significant downward trend in extremity from T0 to T2. Thus, H1c was not supported, suggesting that a 14-day modification of selection mechanisms is insufficient to structurally alter deeply held socio-political attitudes.

**Metacognitive awareness (H1e)**. The AMAS model test revealed a substantial Condition × Time interaction, *F*_(3, 594)_ = 11.85, *p* < 0.001, ${\;\eta }_{p}^{2}$ = 0.056. Experimental group AMAS scores increased significantly from baseline (*M* = 2.85) to post-intervention T2 (*M* = 3.35, *p* < 0.001, and *d* = 0.80). Interestingly, unlike reported content-exposure breadth, this metacognitive awareness persisted at T3 (*M* = 3.28). Experiencing the contrast between reduced personalized recommendation exposure and normal platform use was associated with higher self-reported metacognitive awareness of algorithmic influence, supporting H1e.

**Figure 3:**
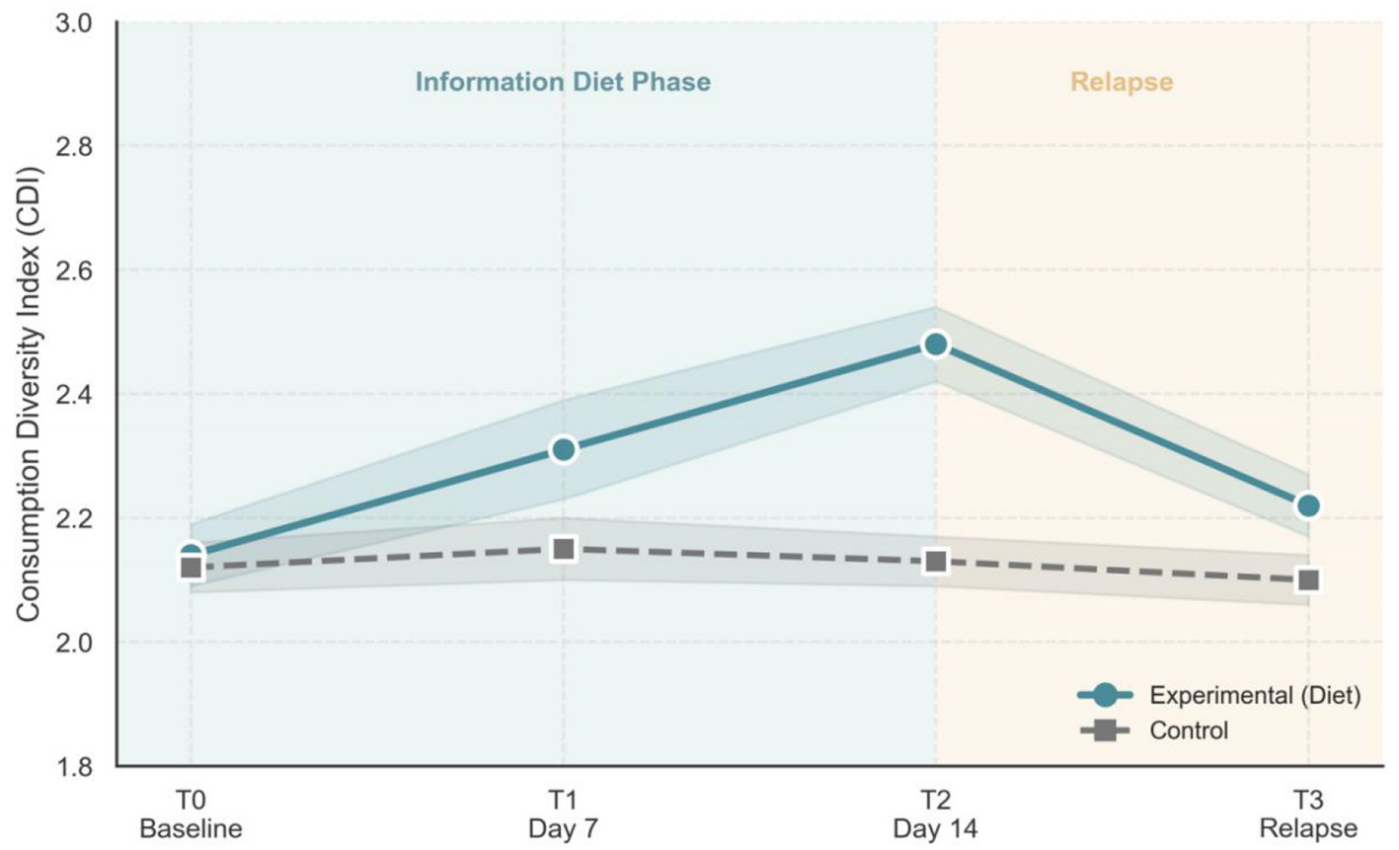
The information diet effect on reported content-exposure breadth over 21 days (H1a and H1d). The solid teal line and dashed gray line represent the mean CDI scores for the experimental and control groups, respectively. Shaded bands around the lines indicate 95% confidence intervals. The light blue background marks the 14-day information diet intervention, while the light orange background marks the 7-day post-intervention readjustment period after personalized feeds were resumed.

**Figure 4:**
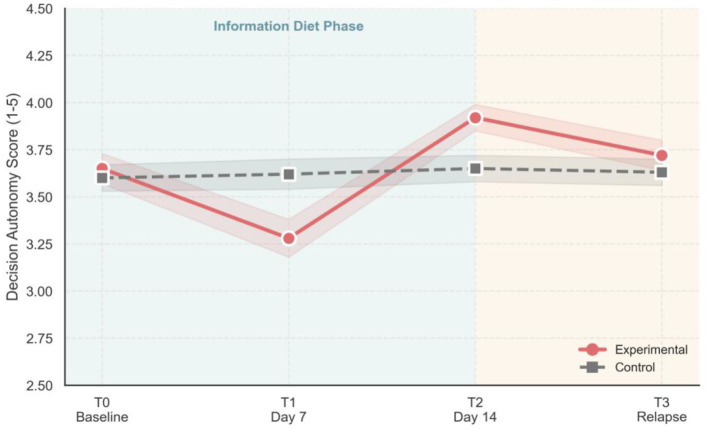
The U-shaped adjustment pattern for decision autonomy (H1b). The experimental group showed a transient drop followed by a significant rebound above baseline. The solid coral line represents the experimental group, and the dashed gray line represents the control. Semi-transparent shaded bands denote 95% confidence intervals around the group means. The background coloration differentiates the active intervention phase (light blue) from the short-term post-intervention readjustment phase (light orange).

**Self-concept clarity**. An exploratory ANOVA on SCC scores yielded no main or interaction effects (all *ps* > 0.30). This null result is consistent with the interpretation that the 14-day intervention was more relevant to state-level preference expression than to durable identity-related constructs. Descriptive statistics for Study 1 are reported in Table 3. The CDI trajectory is shown in Figure 3, and the decision autonomy trajectory is shown in Figure 4.

**Table 3:** Descriptive statistics for primary outcomes over 21 days (Study 1).

Variable	Condition	T0 (Baseline)	T1 (Day 7)	T2 (Day 14)	T3 (Day 21)
Reported content-exposure breadth (CDI)	Experimental	2.14 ± 0.42	2.31 ± 0.45	2.48 ± 0.47	2.22 ± 0.43
	Control	2.12 ± 0.40	2.15 ± 0.42	2.13 ± 0.39	2.10 ± 0.41
Decision autonomy	Experimental	3.65 ± 0.55	3.28 ± 0.58	3.92 ± 0.52	3.72 ± 0.54
	Control	3.60 ± 0.52	3.62 ± 0.54	3.65 ± 0.51	3.63 ± 0.53
Metacognitive awareness (AMAS)	Experimental	2.85 ± 0.65	–	3.35 ± 0.60	3.28 ± 0.62
	Control	2.80 ± 0.62	–	2.82 ± 0.60	2.81 ± 0.61

#### 4.2.3 Process log analysis

**Figure 5:**
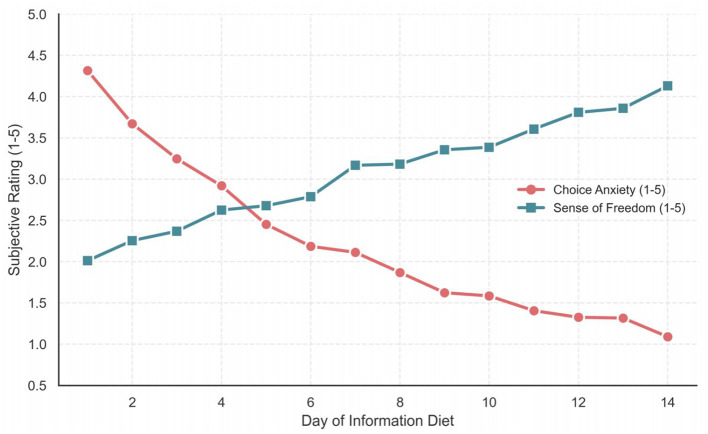
Fourteen-day trajectory of information diet experience based on daily log analysis. The coral line tracks self-reported choice anxiety, demonstrating a rapid decline after early friction. The teal line tracks the self-reported sense of freedom, which increases steadily over the 2-week intervention. Individual opaque markers denote daily group mean scores.

Multi-level modeling (HLM) of the 14-day daily diary data corroborated the discrete survey findings. Daily reports of choice anxiety in the experimental group peaked on Day 3 and declined thereafter (γ = −0.15, *p* < 0.001), broadly corresponding to the U-shaped adjustment pattern in perceived autonomy. The self-reported proportion of actively sought content vs. passively consumed content increased over the 14 days, providing process-level evidence that participants engaged in more active selection during the information diet. This indicator served as a behavioral proxy for attention-related selection, not as a direct measure of visual attention or in-app attentional allocation. Similarly, choice anxiety and active-seeking reports captured local decision friction within the information diet, but they did not provide a full cognitive-effort budget across other digital or non-digital decision domains. Daily process-log averages are summarized in Table 4. The daily process-log trends are shown in Figure 5.

**Table 4:** Daily process log averages across intervention weeks (Study 1 experimental group).

Metric	Week 1 (Days 1–7 M)	Week 2 (Days 8–14 M)	Trend direction
Choice anxiety (1–5)	3.8	2.1	Decreasing (–)
Sense of freedom (1–5)	2.5	3.9	Increasing (+)
Active content seeking (%)	40.5%	75.2%	Increasing (+)

### 4.3 Discussion

Study 1 suggests that reducing exposure to personalized recommendation feeds, while preserving access to digital platforms and social communication, was associated with an increase in reported content-exposure breadth among educated Chinese undergraduate participants. This increase should be read as broader reported exposure across content categories, not as direct evidence that participants intentionally explored every category or developed more durable preferences. This finding addresses an important limitation of conventional digital detox research by targeting personalized feed exposure more specifically than total platform abstinence. At the same time, the intervention should not be interpreted as a fully algorithm-free or substitution-free condition. Daily logs, browser monitoring, and screen-time screenshots provided partial checks on protocol adherence and total platform use, but they could not fully determine whether participants shifted attention to other algorithmically curated mobile environments. Participants may also have encountered social cues in chronological or social-graph feeds, while personalized recommendation cues may have operated together with non-recommendation interface affordances such as autoplay, notifications, infinite scroll, and default feed placement. The present design, therefore, speaks to reduced exposure to user-facing personalized recommendation interfaces, rather than to the isolated causal effect of algorithmic curation alone or to any specific content-based, collaborative-filtering, or hybrid recommendation model. The same caution applies to Layer 1: the present data capture attention-related selection through reported exposure breadth, interface-use logs, and active-seeking reports, but they do not capture visual attention within mobile feeds. Ethically, the intervention should also be understood as a feed-structure modification rather than an information-deprivation protocol, since participants retained access to direct search, subscriptions, official information sources, and interpersonal communication.

The U-shaped trajectory of decision autonomy, together with the process-log data, suggests a short-term adjustment process following the reduction of personalized recommendation exposure. The initial decline in autonomy may reflect habit disruption and increased cognitive-control demands: participants had to actively search, compare, and select content that was previously supplied through personalized feeds. This temporary friction is consistent with the increase in choice anxiety during the early intervention period. By the second week, repeated active selection was accompanied by lower choice anxiety and a stronger sense of self-directed engagement, consistent with Self-Determination Theory's view of autonomy as volitional self-endorsement. At the same time, the intervention may also have affected competence and relatedness. Active search may have changed participants' sense of effectiveness in navigating digital environments, while chronological or social-graph feeds may have altered exposure to peer-generated content and social cues. The autonomy rebound should, therefore, be interpreted as a perceived autonomy pattern within a broader shift in digital need satisfaction, not as evidence of autonomy change independent of competence or relatedness. Moreover, the absence of direct measures of cognitive effort, working memory load, competence satisfaction, relatedness satisfaction, and formal mediation pathways limits this mechanism to a theoretically grounded and process-consistent interpretation rather than a conclusively causal explanation.

However, Study 1 leaves a critical mechanistic question unanswered. While Study 1 suggested that reduced personalized recommendation exposure was associated with changes in reported content-exposure breadth and perceived autonomy, it did not directly identify the cognitive processes involved when users encounter immediate recommendation cues. Furthermore, Study 1 also showed that metacognitive awareness (AMAS) increased after participants experienced reduced personalized recommendation exposure. Does this heightened metacognitive awareness actually protect users from making algorithmically confluent choices when they return to standard platforms? Study 2, therefore, shifts to a controlled behavioral paradigm to examine whether recommendation-label effects differ across speeded and more deliberative decision contexts.

## 5 Study 2: dual-process decision experiment

While Study 1 examined the field-level effects of reduced personalized recommendation exposure, Study 2 used a controlled behavioral paradigm to examine whether recommendation labels exert stronger influence under speeded choice conditions than under deliberative reasoning conditions. Drawing on dual-process theory, we treated the rapid consumer-choice task and the deliberative opinion-formation task as relative processing conditions rather than pure measures of System 1 and System 2 cognition. The purpose was to test whether algorithmic labels have greater behavioral effects when decisions are made quickly and with limited opportunity for comparison, while acknowledging that the present design does not directly measure cognitive load, working memory engagement, or response caution.

### 5.1 Methods

#### 5.1.1 Participants

A new sample of 300 undergraduate students (distinct from Study 1) was recruited. A priori power analysis for repeated-measures ANOVA (*f* = 0.25, α = 0.05, power = 0.80, and four measurements) indicated a minimum requirement of 158 participants; we oversampled to account for potential exclusions due to failed manipulation checks. The final sample consisted of 286 participants (*M*_*age*_ = 21.1, *SD* = 1.6; 58% female) after excluding those who failed attention checks. Participants received course credit or modest financial compensation.

#### 5.1.2 Design

The experiment employed a 2 (Recommendation Label: Present vs. Absent) × 2 (Explanation/Context: None vs. Present) within-subjects design, assessed across two processing conditions: a speeded consumer-choice task and a deliberative opinion-formation task. These tasks were designed to approximate relatively intuitive vs. relatively reflective decision contexts, rather than to provide process-pure measures of System 1 and System 2 cognition.

#### 5.1.3 Materials

**Task A: Rapid consumer decision**. We constructed a simulated e-commerce interface using the jsPsych library. Participants viewed 12 consecutive trials presenting two functionally and financially equivalent products, such as two brands of portable chargers. They were required to make a selection within a 5-s response window. This time window was intended to increase reliance on rapid, intuitive processing by limiting extended comparison and deliberation. We do not treat the 5-s condition as a pure or exhaustive measure of System 1 processing. Rather, it served as a speeded-choice condition that could be contrasted with the deliberative writing task. Reaction time was recorded as a behavioral indicator of decision latency, but the task did not directly measure cognitive load, working memory, or response caution.

1. Baseline: No labels.
2. Algorithmic Label: One product was badged with “Recommended for You.”
3. Explainable AI (XAI): One product was badged with “Recommended based on your browsing history.”
4. Social Proof: One product was badged with “7% of similar users bought this.”

**Task B: Deliberative opinion formation**. To create a relatively more reflective comparison condition, participants read materials concerning three polarizing socio-technical issues, such as implementing mandatory real-name registration on social media. For each issue, they were presented with two equally rigorous 300-word arguments, one supporting and one opposing the issue. In the recommendation-label condition, one argument was prominently flagged with an “Algorithmically Selected Recommendation” badge. Participants were given unlimited time to read the texts and were required to write a 100-word paragraph articulating their own stance. This task was designed to encourage more deliberative processing than the speeded consumer-choice task, although it should not be interpreted as a direct measure of System 2 processing in the absence of cognitive-load or working-memory indicators.

#### 5.1.4 Measures

**Conformity rate:** In Task A, this was the percentage of trials where the participant selected the labeled item over the unlabeled equivalent. In Task B, conformity was operationalized as the semantic alignment between the participant's written response and the position advocated by the labeled text, coded by two blind raters (0 = complete opposition, 100 = complete alignment; inter-rater reliability *r* = 0.88).

**Reaction time (RT):** Captured in milliseconds for Task A choices via jsPsych.

**Cognitive reflection test (CRT):** The standard three-item CRT measured individual differences in reflective cognitive processing and the tendency to override intuitive responses.

**Need for cognition (NFC)**. The 18-item short-form NFC scale measured the intrinsic tendency to engage in and enjoy effortful thinking (α = 0.85). CRT and NFC were included as theoretically relevant but non-exhaustive indicators of reflective disposition. The present design did not include separate measures of impulsivity, need for cognitive closure, susceptibility to social influence, or trait conformity; therefore, the moderation analyses should be interpreted as a partial test of individual differences in algorithmic conformity.

**Algorithmic metacognitive awareness scale (AMAS):** The 12-item scale validated in Study 1 was administered to assess real-time awareness (α = 0.88). Study 2 measures are summarized in Table 5.

**Table 5:** Summary of measures (Study 2).

Measure	Operationalization	Scale source
Conformity rate (Task A)	% selection of labeled item over equivalent	Behavioral
Decision RT (Task A)	Time in ms	Behavioral
Conformity score (Task B)	0%−100% semantic alignment with labeled text	Blind coded
Cognitive reflection (CRT)	three-item mathematical overrides	
Need for cognition (NFC)	18-item intrinsic thought engagement	

#### 5.1.5 Procedure

The experiment was conducted entirely online via a bespoke integration of Qualtrics and jsPsych. Following informed consent, participants completed the CRT and NFC scales. They then completed Task A and Task B (counterbalanced). Following the cognitive tasks, participants completed the AMAS, a manipulation check confirming visibility of the recommendation labels, and a demographic questionnaire. Because the recommendation labels were artificially generated, participants underwent a funnel debriefing protocol at the study's conclusion. This included a suspicion probe to identify and exclude any participants who accurately guessed the explicit intent of varying the algorithmic tags (i.e., demand characteristics), followed by a comprehensive explanation of the deceptive manipulation.

### 5.2 Results

#### 5.2.1 Manipulation check

Analyses confirmed that 95.3% of participants successfully identified the presence of recommendation labels during the tasks. Participants who failed this check (*n* = 14) were excluded from the primary analyses, yielding the final sample of 286.

#### 5.2.2 Task A results: speeded consumer choice

Conformity rates in the rapid consumer choice task were analyzed using a repeated-measures ANOVA across the four label conditions. A significant main effect of Condition emerged, *F*_(2.8, 802.4)_ = 48.62, *p* < 0.001, and ${\;\eta }_{p}^{2}\;=$ 0.145.

**Figure 6:**
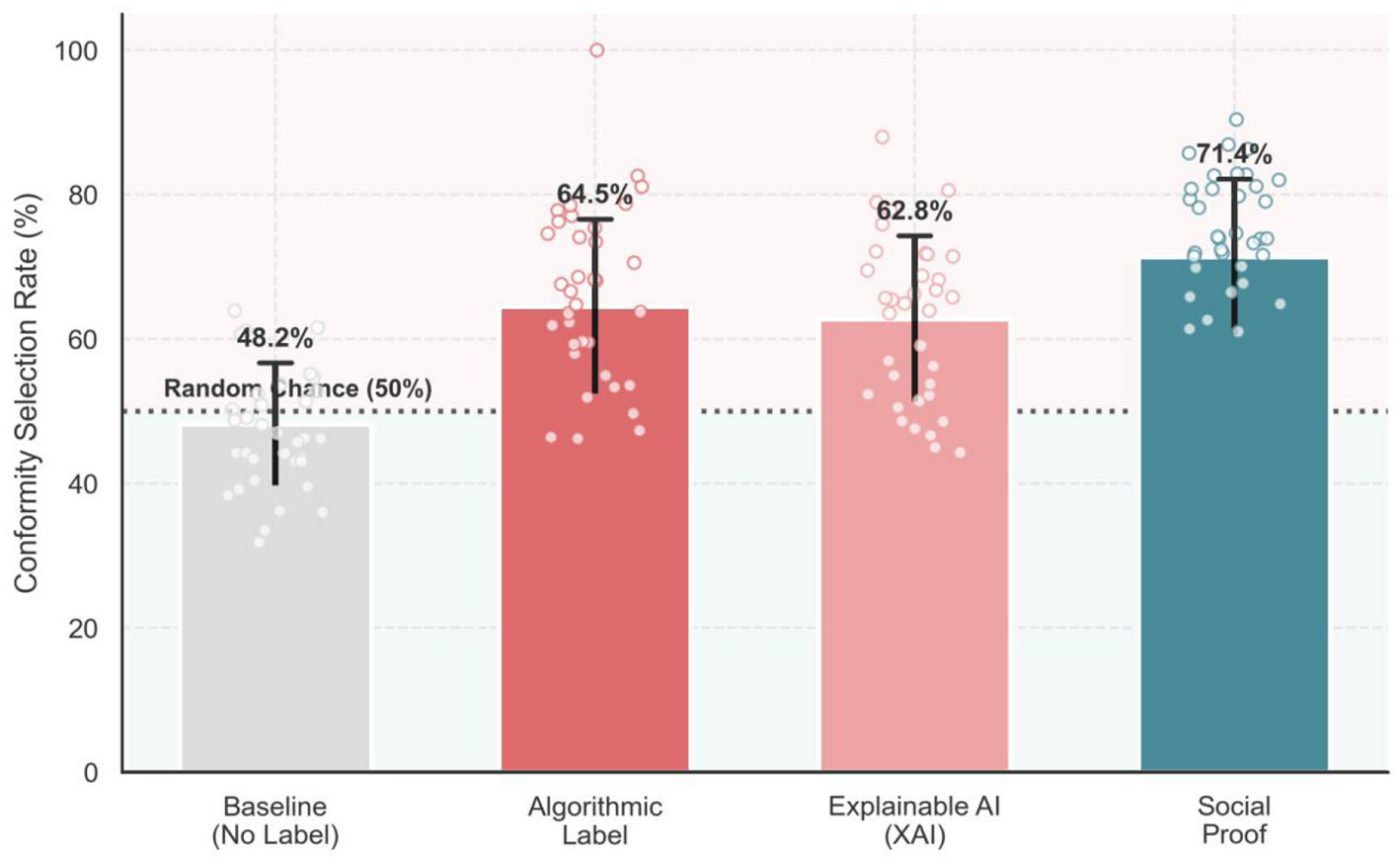
Conformity rates across Task A conditions. Colored bars represent the mean conformity selection rate for each experimental label, with vertical black error bars indicating standard deviations. The overlaid semi-transparent white scatter dots represent the distribution of individual participant conformity rates, indicating underlying data dispersion. The horizontal dotted line situated at 50% represents the baseline probability of random chance selection between two equivalent options.

Planned contrasts revealed that compared to the Baseline condition (*M* = 48.2%, *SD* = 8.5%, reflecting random choice between equivalent items), the simple Algorithmic Label condition significantly increased conformity (*M* = 64.5%, *SD* = 12.1%, *p* < 0.001, and *d* = 0.81), supporting H2a. Interestingly, the Explainable AI (XAI) label (*M* = 62.8%, *SD* = 11.5%) did not significantly reduce conformity compared to the opaque label (*p* = 0.18), contrary to H2b's prediction of protective transparency. The strongest conformity effect was observed in the Social Proof condition (*M* = 71.4%, *SD* = 10.8%), which was significantly higher than the standard algorithmic label (*p* < 0.001, *d* = 0.45), supporting H2c. Reaction time analysis showed that selections in all labeled conditions were significantly faster than baseline (*p* < 0.01), suggesting reduced decision latency in labeled conditions. Task A descriptive statistics are shown in Table 6. The conformity selection rates across conditions are shown in Figure 6.

**Table 6:** Task A descriptive statistics: speeded consumer choice.

Condition	Conformity rate (*M* ±*SD*)	Reaction Time (*M* ±*SD*, ms)
Baseline (no label)	48.2% ± 8.5%	3,240 ± 540
Algorithmic label	64.5% ± 12.1%	2,450 ± 480
Explainable AI (XAI)	62.8% ± 11.5%	2,510 ± 490
Social proof	71.4% ± 10.8%	2,280 ± 410

#### 5.2.3 Task B results: deliberative opinion formation

For the deliberative writing task, compliance with the algorithmic recommendation was assessed via the blinded semantic alignment scores. A paired-samples *t*-test indicated that the presence of a recommendation label produced a statistically significant, but practically marginal, increase in viewpoint alignment [*M*_*labeled*_ = 54.2%, *SD* = 14.5% vs. *M*_*baseline*_ = 49.8%, *SD* = 15.1%; *t*_(285)_ = 2.45, *p* = 0.015, *d* = 0.14].

**Figure 7:**
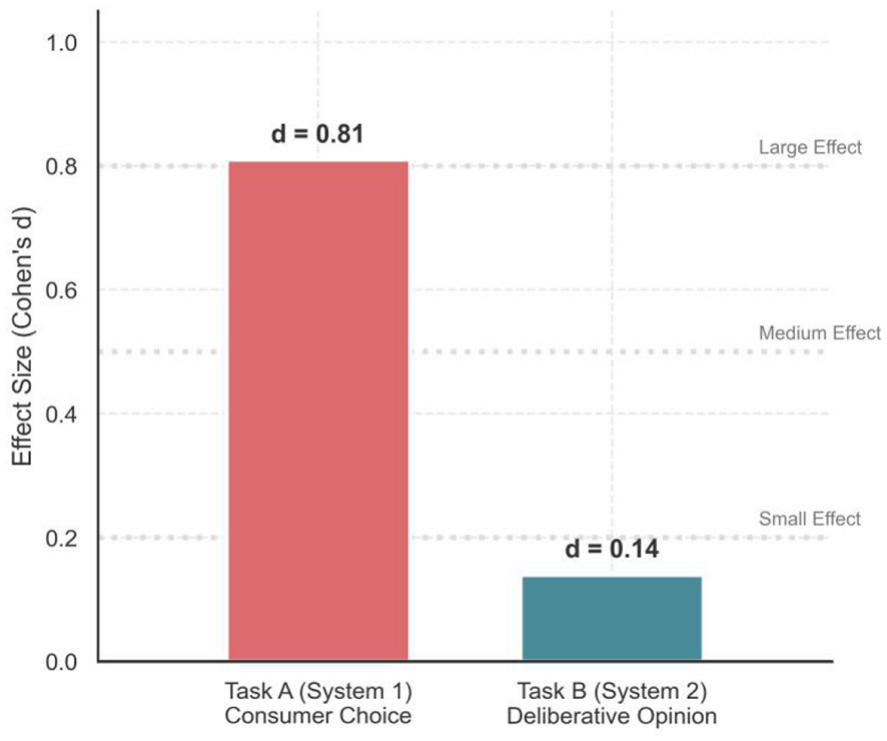
Comparing algorithmic effect size (Cohen's *d*) in speeded choice and deliberative opinion-formation tasks. The coral bar shows the behavioral effect in the speeded consumer-choice task, whereas the teal bar shows the smaller effect in the deliberative opinion-formation task. Horizontal dotted lines indicate standard statistical benchmarks for small (*d* = 0.2), medium (*d* = 0.5), and large (*d* = 0.8) effect sizes.

Critically, a 2 (Task: Speeded Choice vs. Deliberative Opinion Formation) × 2 (Label: Absent vs. Present) interaction analysis comparing normalized conformity effect sizes revealed a significant interaction, *F*_(1, 285)_ = 112.4, *p* < 0.001, ${\;\eta }_{p}^{2}$ = 0.28. The behavioral influence of algorithmic badges in the speeded consumer-choice task (Cohen's *d* = 0.81) was larger than its influence in the deliberative opinion-formation task (Cohen's *d* = 0.14). This pattern supports H2a by showing that recommendation labels produced a substantially larger behavioral effect in the speeded consumer-choice task than in the deliberative opinion-formation task. However, because the study did not directly measure cognitive load, working memory, or response caution, the result should be interpreted as evidence of task-dependent differences in algorithmic conformity rather than as definitive proof of distinct cognitive-system capture. The task-level effect sizes are shown in Figure 7.

#### 5.2.4 Moderation analysis (cognitive reflection)

**Figure 8:**
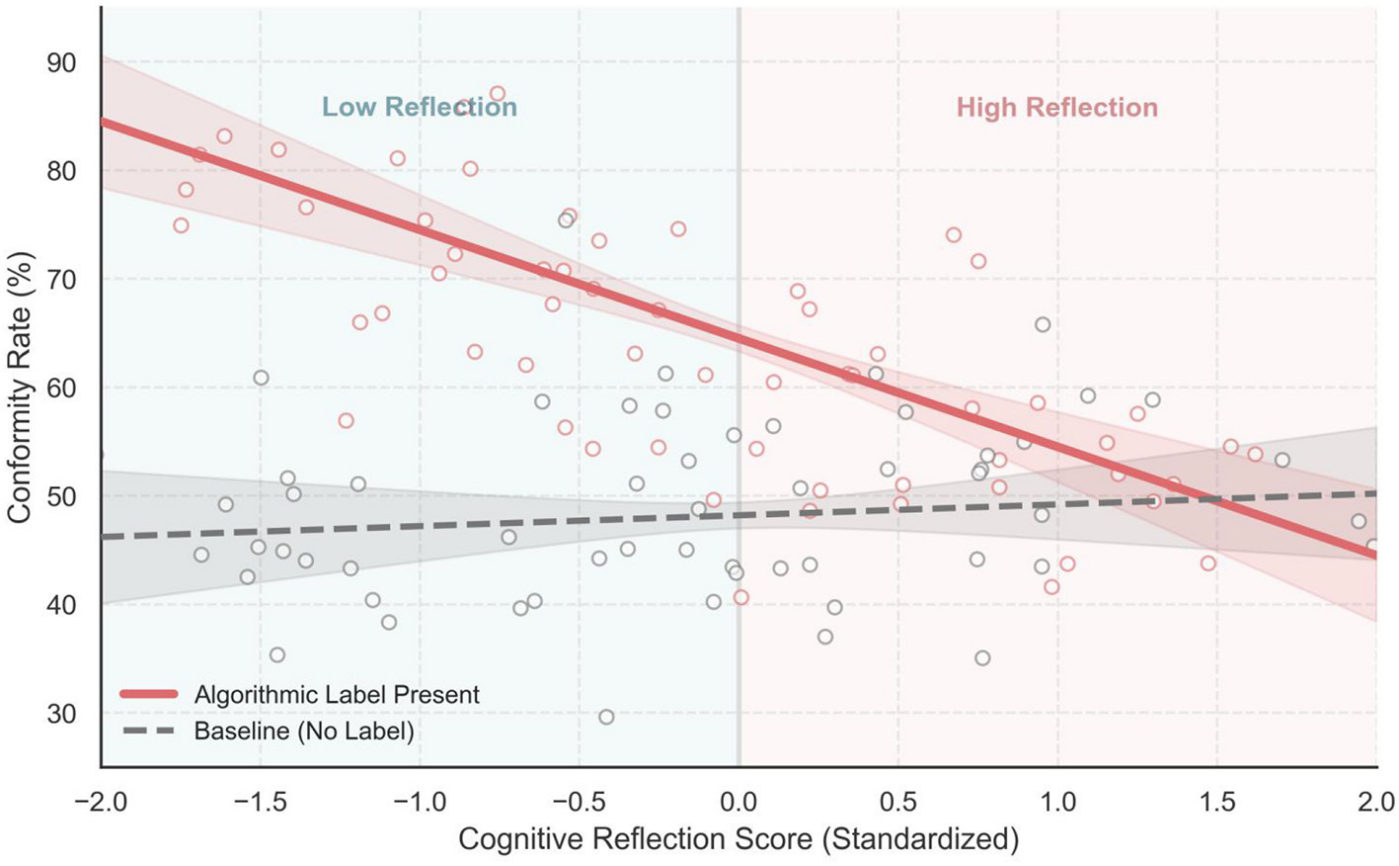
CRT moderates algorithmic conformity. Higher CRT scores were associated with weaker conformity to algorithmic labels in the speeded-choice task. The solid coral line and dashed gray line represent linear regression fits for the algorithmic label condition and baseline condition, respectively, with shaded bands representing 95% confidence intervals.

To test H2d, we utilized the PROCESS macro (Model 1) with Task A conformity rate as the dependent variable, presence of the algorithmic label as the predictor, and CRT score as the continuous moderator. The interaction term was highly significant (Δ*R*^2^ = 0.05, *p* < 0.001). Simple slopes analysis indicated that, for participants with low CRT scores (−1 *SD*), the algorithmic label substantially increased conformity (*b* = 21.4%, *p* < 0.001). For participants with high CRT scores (+1 *SD*), the effect of the algorithmic label was no longer statistically significant (*b* = 3.1%, *p* = 0.22). Need for Cognition showed a similar but weaker moderating pattern. Higher cognitive reflection was, therefore, associated with weaker conformity to algorithmic labels in the speeded-choice task. This moderation pattern should not be interpreted as a complete individual-difference model of algorithmic conformity. It indicates that reflective disposition mattered in the present task, while leaving open the possibility that impulsivity, need for cognitive closure, social influence susceptibility, and trait conformity may explain additional heterogeneity in responses to recommendation cues. The moderating role of CRT is shown in Figure 8. The task-level effects and CRT moderation results are summarized in Table 7.

**Table 7:** Summary of algorithmic influence by cognitive task and CRT moderator (Study 2).

Cognitive condition	Baseline (*M*)	Labeled (*M*)	Effect Size (*d/b*)
Task mode			
Task A (speeded choice)	48.2%	64.5%	*d* = 0.81^***^
15.6-7.5,-14.1242ptTask B (deliberative opinion formation)	49.8%	54.2%	*d* = 0.14^*^
Moderator (Task A)			
Low CRT (−1 *SD*)	47.2%	68.6%	*b* = 21.4%^***^
High CRT (+1 *SD*)	49.2%	52.3%	*b* = 3.1%^ns^

^*^p < 0.05,

^***^p < 0.001; ns, not significant.

#### 5.2.5 The literacy paradox (metacognitive disconnect)

Addressing H2e, we correlated participants' self-reported Algorithmic Metacognitive Awareness Scale (AMAS) scores with their behavioral conformity rates in Task A. Pearson correlation analysis revealed a non-significant and negligible association (*r* = −0.04, *p* = 0.48). Participants in the highest quartile of metacognitive awareness were behaviorally indistinguishable from those in the lowest quartile under speeded recommendation-label conditions. This pattern supports H2e and is consistent with the proposed literacy paradox: self-reported metacognitive awareness of algorithmic influence did not significantly predict behavioral resistance in the speeded-choice task. However, this finding should be interpreted narrowly. It does not imply that algorithmic awareness is irrelevant across users or contexts. Rather, it suggests that awareness alone was insufficient to predict resistance in this task, and that its effect may depend on additional traits such as impulsivity, need for cognitive closure, susceptibility to social influence, or trait conformity.

### 5.3 Discussion

Study 2 provides controlled behavioral evidence that recommendation labels exert stronger influence under speeded choice conditions than under deliberative opinion-formation conditions. This pattern is consistent with a dual-process interpretation, in which algorithmic cues are more influential when users have limited time for comparison and reflective evaluation. However, the simulated label paradigm isolates only one visible recommendation cue within a simplified interface. Real-world platforms combine recommendation labels with additional friction-reducing affordances, including feed ranking, autoplay, infinite scroll, notifications, and default placement. Therefore, the present paradigm should not be interpreted as directly measuring System 1 or System 2 processes, nor as estimating the independent effect of algorithmic curation in full-scale platform environments. Without independent indicators of cognitive load, working memory, response caution, or attentional allocation, the findings are best understood as task-level behavioral evidence rather than direct evidence of cognitive-system capture.

Two patterns are noteworthy. First, the XAI label did not significantly reduce conformity relative to the opaque recommendation label, suggesting that brief explanatory information may be insufficient to alter speeded choices in this task. Second, AMAS scores were not significantly associated with behavioral conformity, supporting the proposed literacy paradox as a self-report–behavior dissociation. This pattern suggests that metacognitive awareness alone may not reliably predict resistance to recommendation cues under speeded-choice conditions. However, the literacy paradox should be interpreted as task-specific rather than trait-general. The present study included CRT and NFC as indicators of reflective disposition, but did not measure impulsivity, need for cognitive closure, susceptibility to social influence, or trait conformity. These unmeasured traits may condition whether algorithmic awareness translates into behavioral resistance. Moreover, the absence of direct measures of cognitive load and response caution limits this interpretation to behavioral task performance rather than underlying cognitive mechanism.

## 6 General discussion

### 6.1 Summary of findings

The present research examined how personalized recommendation systems are associated with reported content-exposure breadth, perceived decision autonomy, and algorithmic conformity among educated Chinese undergraduate participants. Across two complementary behavioral studies, the findings provide preliminary support for selected components of the ACC framework. Study 1 suggests that reducing exposure to personalized recommendation feeds is associated with increased reported content-exposure breadth and a short-term U-shaped trajectory in perceived decision autonomy. Study 2 indicates that recommendation labels exert stronger behavioral effects under speeded consumer-choice conditions than under deliberative opinion-formation conditions.

Study 1 showed that reducing exposure to personalized recommendation feeds was associated with higher reported content-exposure breadth, followed by a short-term return toward baseline after participants resumed normal personalized feed use. Interpreted together with active-seeking logs, this pattern suggests broader exposure accompanied by more self-directed selection, but it does not establish wholly intentional exploration, durable trait-preference change, stable re-colonization, or identity reconstruction. It should, therefore, be interpreted as evidence associated with reduced exposure to user-facing personalized recommendation environments, not as the isolated effect of algorithmic curation separated from interface affordances. The hypothesis-testing results are summarized in Table 8.

**Table 8:** Summary of hypotheses testing.

Hypothesis	Description	Result
Study 1: Information diet		
H1a	CDI indicates increased reported content-exposure breadth during the information diet	Supported
H1b	Autonomy follows a U-shaped adjustment pattern	Supported
H1c	Reduced political extremity	Not Supported
H1d	CDI shows a short-term return toward baseline after personalized feeds resume	Supported
H1e	AMAS increases post-intervention	Supported
Study 2: Dual-process decision		
H2a	Conformity is stronger in speeded choice than deliberative opinion formation	Supported
H2b	XAI transparency reduces conformity	Not Supported
H2c	Social proof generates highest conformity	Supported
H2d	CRT moderates algorithmic influence	Supported
H2e	AMAS is weakly associated with behavioral resistance	Supported

### 6.2 Theoretical contributions

This research offers three main contributions to the behavioral sciences and human-computer interaction literature.

First, it provides an initial behavioral test of selected components of the ACC framework. Previous scholarship has documented filter bubbles and narrowed informational exposure (Pariser, 2011). The present findings extend this discussion by showing that reduced personalized recommendation exposure was associated with short-term changes in reported content-exposure breadth and perceived autonomy. Interpreted alongside active-seeking logs, these results are consistent with short-term state-level preference expression, but the 1-week follow-up does not demonstrate durable trait-preference change, stable re-colonization, or identity reconstruction.

Second, by applying a dual-process perspective to recommendation cues within digital choice architecture, the study shows that recommendation labels may have stronger behavioral effects in speeded, low-deliberation contexts than in more reflective contexts. This finding does not establish direct cognitive-system capture or the independent effect of algorithmic ranking alone. Instead, it suggests that recommendation cues may be most consequential when embedded in interfaces that encourage rapid, low-friction decisions. The contribution, therefore, lies in identifying a task-dependent behavioral pattern that future studies can test more directly by manipulating recommendation cues and interface affordances separately while incorporating cognitive-load measures, response-caution modeling, eye-tracking, or psychophysiological indicators.

Third, the observed dissociation between AMAS scores and behavioral conformity contributes to research on algorithmic literacy by showing that self-reported awareness did not reliably predict resistance to recommendation cues in the present speeded-choice context. This finding suggests that digital literacy research should distinguish metacognitive awareness from behavioral resistance, while also considering how awareness interacts with broader individual differences such as impulsivity, closure motivation, susceptibility to social influence, and trait conformity.

### 6.3 Practical implications

The practical implications of these findings should be interpreted cautiously. The results suggest that transparency labels alone may be insufficient to reduce conformity under speeded-choice conditions, whereas broader changes in feed structure and interface friction may be more closely associated with perceived autonomy and reported content-exposure breadth. However, the current data do not establish which specific platform intervention would be most effective across populations or platforms. Therefore, design recommendations such as providing accessible chronological modes, increasing user control over recommendation settings, or introducing friction before high-stakes choices should be understood as theoretically informed implications rather than directly tested prescriptions.

In educational contexts, algorithmic literacy may benefit from being combined with behavioral exercises that help students observe how feed structures influence their own choices. Guided information-diet activities could be explored as one possible pedagogical tool, although the present study does not test curriculum-level outcomes. Similarly, the moderating role of CRT suggests that reflective decision habits may be relevant to algorithmic conformity, but educational interventions designed to improve such habits require direct evaluation. Any educational or platform-based information-diet intervention should preserve access to essential information and social communication, and should include mechanisms for participants to report missed information, discomfort, or excessive effort burden.

### 6.4 Policy implications

From a policy perspective, the findings are relevant to ongoing debates about recommendation-system transparency and user control. However, the present study does not directly evaluate regulatory frameworks, legal compliance mechanisms, or platform-level default settings. References to policy instruments should, therefore, be treated as contextual discussion rather than empirical validation of any specific regulation. Future research should directly test whether opt-in recommendation settings, non-profiling defaults, or user-selected feed modes produce measurable benefits across different demographic groups, platform architectures, and cultural contexts, while also monitoring potential costs such as missed information, reduced accessibility, or increased search burden. Such possibilities should be treated as hypotheses for future policy and platform-design research rather than conclusions directly established by the present studies.

### 6.5 Limitations and future directions

Several limitations should be considered when interpreting the present findings. First, the information diet was implemented in naturalistic digital environments and, therefore, could not create a fully algorithm-free or substitution-free condition. Although daily logs, browser monitoring, screen-time screenshots, and perceived algorithmic disengagement items provided convergent evidence that the intervention reduced exposure to personalized recommendation interfaces, these indicators could not fully decompose mobile in-app behavior by feed type. Participants may have encountered residual personalized content within mobile applications or shifted attention to other algorithmically curated platforms not fully captured by the monitoring tools. Accordingly, the Study 1 findings should be interpreted as evidence associated with reduced personalized recommendation-interface exposure, not as the isolated causal effect of algorithmic curation alone. Future studies should combine platform-level data donation, privacy-preserving mobile telemetry, passive sensing, and cross-platform usage logs to distinguish reduced recommendation exposure from compensatory platform switching.

Second, the present measurements captured behavioral and self-reported indicators rather than direct attentional, cognitive-load, or physiological processes. Layer 1 of the ACC framework was operationalized as attention-related behavioral selection, indexed through feed-structure exposure, browser-logged interface use, active-seeking reports, and reported content-exposure breadth. However, the study did not collect gaze dwell time, scroll depth, fixation patterns, screen recordings, psychophysiological responses, or neural measures. Similarly, the Consumption Diversity Index captured reported exposure breadth across content categories, not verified intentional exploration, direct visual attention, or state-level preference expression. Choice anxiety and active-seeking logs provided useful process indicators, but they did not constitute a full cognitive-effort budget across digital and non-digital decision domains. Future research should incorporate eye-tracking, scroll-depth records, ecological momentary assessment, mental fatigue ratings, response-caution modeling, and psychophysiological measures to test attentional and cognitive-effort mechanisms more directly.

Third, the motivational and technical mechanisms underlying the observed effects require more precise separation. The present studies treated perceived decision autonomy as the focal Self-Determination Theory outcome, but competence and relatedness were not measured as separate dependent variables. This limits the interpretation of the U-shaped autonomy pattern, because active search may have changed participants' perceived competence, while chronological and social-graph feeds may have altered relatedness through peer-generated content, social cues, or network-based visibility. In addition, the studies did not experimentally distinguish content-based, collaborative-filtering, and hybrid recommender architectures. Nor did they separate algorithmic ranking from non-algorithmic interface affordances such as autoplay, infinite scroll, notifications, haptic feedback, one-click actions, or default feed placement. Future studies should measure all three SDT needs and use factorial designs that independently manipulate recommendation logic and interface affordances to identify their separate and interactive effects on perceived autonomy, state-level preference expression, and recommendation-label conformity.

Fourth, the temporal and population scope of the findings remains limited. Study 1 used a 14-day intervention with a 1-week follow-up, which is sufficient for observing short-term adjustment but not for establishing durable re-colonization, state-level preference expression, or identity-level transformation. The T3 return toward baseline should, therefore, be interpreted as short-term post-intervention readjustment rather than permanent relapse. Layer 3 of the ACC framework remains theoretical in the present manuscript and requires longer longitudinal evidence. In addition, both studies relied on educated Chinese undergraduate participants, limiting generalizability to broader Generation Z populations, non-student groups, older adults, and users in different cultural or platform ecosystems. Future research should use longer follow-up periods, more diverse samples, and cross-cultural designs to examine whether the observed behavioral patterns persist across demographic and technological contexts.

Fifth, the individual-difference model and intervention-risk assessment were necessarily incomplete. Study 2 included CRT and NFC as indicators of reflective disposition, but did not measure impulsivity, need for cognitive closure, susceptibility to social influence, trait conformity, or broader motivational profiles. The literacy paradox should, therefore, be interpreted narrowly: self-reported algorithmic awareness did not predict behavioral resistance in the present speeded-choice task, but awareness may interact with other personality and motivational traits in different contexts. Moreover, although the information diet protocol was designed to preserve access to essential information, direct search, subscriptions, official sources, and interpersonal communication, future research should include more formal adverse-event monitoring to assess missed urgent information, accessibility costs, or excessive search burden. Taken together, these limitations suggest that the ACC framework should be treated as a behavioral foundation for future multimethod research rather than as a complete causal account of neural, attentional, motivational, or identity-level change.

## 7 Conclusions

The digital architecture of contemporary platforms increasingly relies on behavioral prediction and personalized curation. This manuscript introduced the Algorithmic Cognitive Colonization framework and provided an initial behavioral test of selected attentional and state-preference processes. Across two studies with educated Chinese undergraduate participants, reduced exposure to personalized recommendation feeds was associated with higher reported content-exposure breadth and changes in perceived autonomy, while recommendation labels exerted stronger effects under speeded-choice conditions than under deliberative opinion formation. These findings do not establish direct neural mechanisms, durable identity change, or universal effects across Generation Z. Rather, they suggest that safeguarding cognitive autonomy requires attention not only to users' knowledge about algorithms, but also to the choice environments in which digital preferences are expressed.

## Data Availability

The data presented in this study are available from the corresponding author upon reasonable request.
